# Association between systemic immune-inflammation index and cataract among outpatient US adults

**DOI:** 10.3389/fmed.2024.1469200

**Published:** 2024-09-18

**Authors:** Jin Huang, Hongjiang Wu, Fang Yu, Fangkun Wu, Chen Hang, Xiaoya Zhang, Yiting Hao, Hao Fu, Hongting Xu, Rong Li, Ding Chen

**Affiliations:** National Clinical Research Center for Ocular Diseases, Eye Hospital, Wenzhou Medical University, Wenzhou, China

**Keywords:** cataract, systemic immune-inflammation index, cross-sectional study, National Health and Nutrition Examination Survey, outpatient US adults

## Abstract

**Background:**

While several studies have noted a higher SII correlates with multiple diseases, research on the association between SII and cataract remains limited. Our cross-sectional study seeks to examine the association between SII and cataract among outpatient US adults.

**Methods:**

This compensatory cross-sectional study utilized NHANES data from 1999 to 2008 cycles, conducting sample-weighted multivariate logistic regression and stratified analysis of subgroups.

**Results:**

Among 11,205 adults included in this study (5,571 [46.2%] male; 5,634 [53.8%] female), 2,131 (15.2%) had cataract and 9,074 (84.8%) did not have cataract. A fully adjusted model showed that SII higher than 500 × 10^9^/L was positively correlated with an increased risk of cataracts among women (OR, 1.27; 95% CI, 1.02–1.59) (*p* = 0.036). However, no difference was found in the men subgroup, and there was no significant interaction between SII and sex.

**Conclusion:**

Our results indicated that a SII higher than 500 × 10^9^/L was positively correlated with an increased risk of cataracts in women. This study is the first to specifically investigate the impact of a high SII on cataract risk in outpatient adults in the United States. By effectively addressing inflammation, it is possible to mitigate cataract progression and significantly enhance patient outcomes.

## 1 Introduction

Cataract continues to pose a significant global concern for vision impairment and blindness, especially among the elderly population (1–3). In the United States, its prevalence rises from 24.4% in individuals aged 40 and above to over 50% in those aged 75 and older, predominantly due to aging (4). Risk factors such as smoking and alcohol contribute to its development (5–7). Cataract surgery is highly effective, but accessibility is often limited by economic challenges. This issue is particularly pronounced in developing countries (8). Addressing these modifiable risk factors is essential for reducing the health and socioeconomic burden of cataracts.

Research indicates that the SII is associated with pseudophakic cystoid macular edema (PCME) in patients without risk factors after uneventful phacoemulsification cataract surgery, suggesting that SII could serve as a predictive biomarker for PCME, thereby enhancing clinical assessment and risk stratification (9). Similarly, a study in Suzhou, China, found that while SII does not directly correlate with presenting visual impairment (PVI), uncorrected refractive error and cataracts remain the leading causes of PVI, with significant associations linked to older age and elevated fasting blood glucose (10). Furthermore, evidence from another study shows that a pro-inflammatory diet, as quantified by the dietary inflammatory index (DII), is associated with an increased risk of cataract and age-related macular degeneration (AMD), with the neutrophil-lymphocyte ratio (NLR) and other inflammatory markers mediating these effects (11).

In the human body’s systemic inflammatory system, immune cells play a crucial role in various diseases. Researchers have found that the combined counts of lymphocytes, neutrophils, and platelets in peripheral blood can provide a more accurate predictor of inflammatory status. Specifically, the SII is derived from these three types of circulating immune cells (12, 13). Recent studies suggest that systemic inflammation may contribute to photoreceptor cell death in patients with Retinitis Pigmentosa (RP). The NLR, calculated by dividing the total count of neutrophils by that of lymphocytes in a peripheral blood sample, has been proposed as a marker of systemic inflammation and a poor prognostic indicator for various chronic diseases. Additionally, the NLR is emerging as a potential inflammatory marker for several ocular conditions, including keratoconus, retinal vein occlusion (RVO), AMD, and dry eye (14). Cataracts, characterized by the clouding of the eye’s lens, lead to progressive visual impairment and are a major cause of blindness globally. The core features of cataracts include lens opacity, reduced light transmission, and compromised visual acuity. Inflammation and immune responses have been increasingly recognized as significant factors in cataract development. Chronic inflammation can induce oxidative stress and promote lens protein modification, which contributes to lens opacity (15). Additionally, immune responses involving cytokines and inflammatory mediators have been implicated in the cataractogenesis process. Given these insights, the SII, which integrates lymphocyte, neutrophil, and platelet counts, may offer a valuable tool for assessing systemic inflammation’s role in cataract formation.

The SII was initially identified as a prognostic marker for conditions such as kidney stones and hepatic steatosis (16, 17). However, its impact on cataracts within the outpatient U.S. population and its prognostic value for cataracts are not yet well established. In this cross-sectional study utilizing NHANES data from 1999 to 2008 cycles, we aimed to investigate the association between SII and cataract among outpatient US adults.

## 2 Methods

### 2.1 Data sources

Information on cataract was only provided in the NHANES 1999–2004 cycles for individuals aged 20 years or older and in the 2005–2008 cycles for individuals aged 50 years or older. In this cross-sectional study, deidentified data for participants aged 50 years or older were extracted from the NHANES 1999–2008 cycles. This study followed the Strengthening the Reporting of Observational studies in Epidemiology (STROBE) reporting guideline. Data were analyzed from March to July 2024.

### 2.2 Study design and population

**Figure 1:**
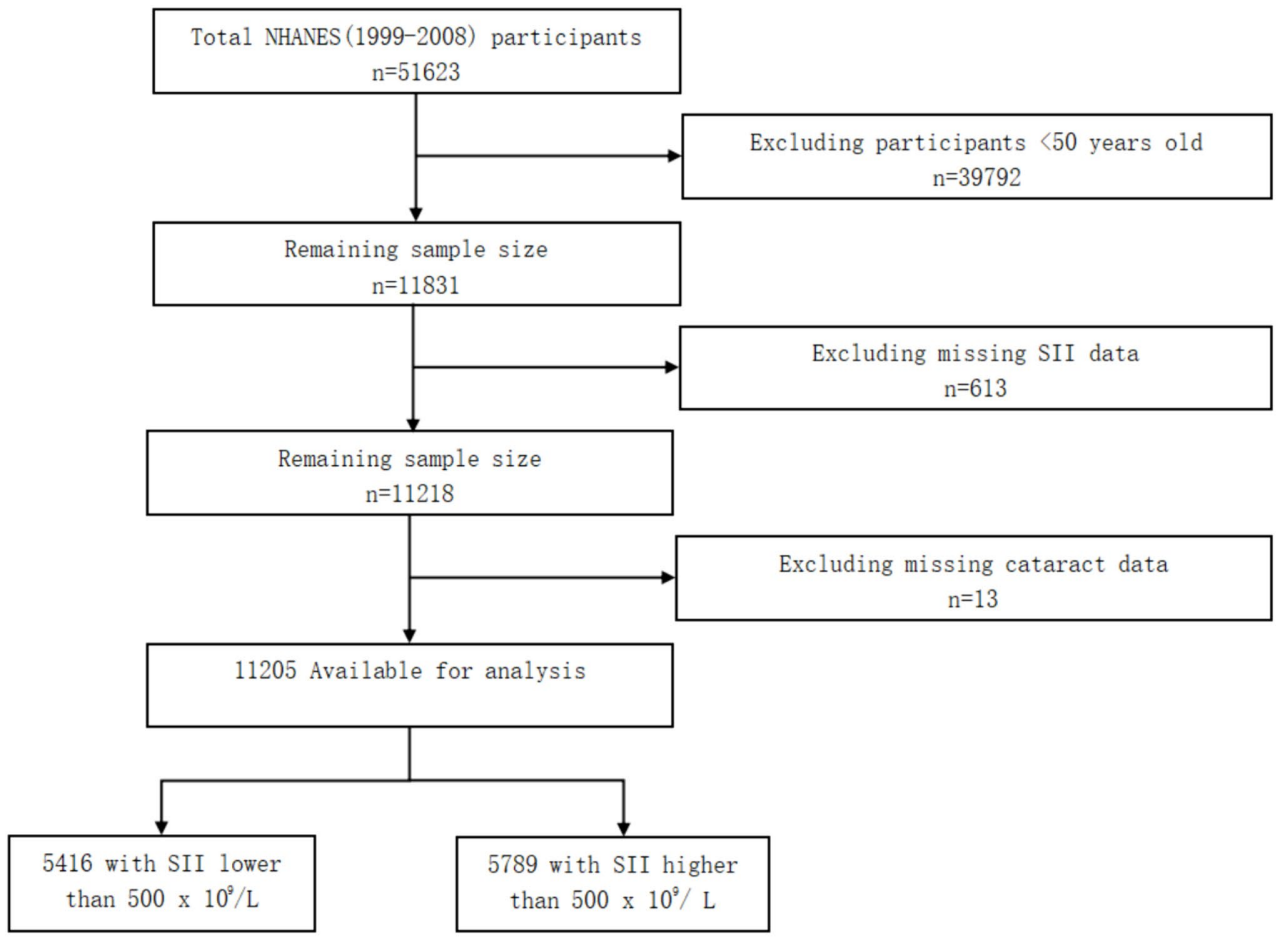
Flow diagram of the screening and enrollment of study participants.

Because information on cataract was only provided in the NHANES 1999–2008 cycles for adults aged 50 years or older, we selected a total of 11,831 adults within this age range. Moreover, we excluded 613 participants who had missing SII data, 13 participants who had missing cataract data. Finally, a total of 11,205 participants were involved. Of the 11,205 participants, there were 5,416 participants with SII lower than 500 × 10^9^/L and 5,789 participants with SII higher than 500 × 10^9^/L (Figure 1).

### 2.3 Assessment of SII and cataract

SII was defined as (platelet x neutrophil)/lymphocyte (18–20). Throughout the analysis, an SII cutoff of 500 × 10^9^/L was applied.

Cataract diagnosis was based on participants’ self-report of having undergone cataract surgery, as indicated by a positive response to the question “Have you ever had a cataract operation?” (VIQ070:1999–2002; VIQ071:2003–2008), with response options of “yes” or “no.” A positive response was considered indicative of a cataract (4, 21).

### 2.4 Covariates

According to previous studies concerning cataract, potential confounding factors studied in the current work included: sociodemographic variables (age, sex, race and ethnicity, family income, educational level, and marital status), NHANES cycles, BMI, lifestyle factors (alcohol drinking status, smoking status), and comorbidities (including hypertension, hyperlipidemia, and diabetes mellitus) (4, 21). Sociodemographic variables, lifestyle factors, and comorbidities were drawn from self-reported questionnaires.

### 2.5 Statistical analysis

Our analyses utilizing NHANES data from 1999 to 2008 cycles considered the complex sampling design and sampling weights and the sampling weight was calculated using the following formula: fasting subsample 10-year mobile examination center (MEC) weight = fasting subsample 2-year MEC weight/5 (22, 23). Moreover, we used quartiles to describe the continuous variables with non-normal distribution and described the categorical variables using unweighted frequency and weighted percentage in this cross-sectional study.

If the VIF was 5 or higher, it meant there was multicollinearity present (22). To investigate the association between SII and cataract, logistic regression models were established. Model 1 was adjusted for sociodemographic variables and NHANES cycles. Model 2 was adjusted for sociodemographic variables, NHANES cycles, BMI, lifestyle factors (alcohol drinking status, smoking status), and comorbidities (including hypertension, hyperlipidemia, and diabetes mellitus). Finally, we conducted a subgroup analysis on gender to assess the possible influence of sex on the association between SII and cataract.

We used R for all the statistical analyses and we considered a significance level of *p* < 0.05 to show that the results were statistically significant.

## 3 Results

There were 11,205 participants included in this study. 39,792 participants <50 years old were excluded, and 613 participants were excluded for missing SII data. After removing 13 participants missing cataract data, 11,205 participants were finally enrolled. Participants with SII levels higher than 500 × 10^9^/L were found to have a higher prevalence of being non-Hispanic White compared to those with SII levels lower than 500 × 10^9^/L (*p* < 0.001). Additionally, we have noted that individuals with higher SII levels are more likely to be current smokers (defined as having smoked at least 100 cigarettes in their lifetime and continuing to smoke), with statistical significance (*p* < 0.001) (see Table 1).

**Table 1:** Characteristics of participants in the NHANES 1999–2008 cycles.

Characteristic	Participants	SII (10^9^/L)		*p*-value
	Total (*N* = 11,205)	< 500 (*N* = 5,416)	≥ 500 (*N* = 5,789)	
Age (median [IQR])	62.00 [54.00, 71.00]	61.00 [54.00, 70.00]	62.00 [54.66, 72.00]	0.002
BMI (median [IQR])	27.90 [24.52, 31.93]	27.86 [24.66, 31.80]	27.93 [24.44, 32.04]	0.914
Sex				0.135
Male	5,571 (46.2)	2,730 (47.1)	2,841 (45.4)	
Female	5,634 (53.8)	2,686 (52.9)	2,948 (54.6)	
Marital status				<0.001
Married	6,527 (66.6)	3,243 (61.1)	3,284 (62.4)	
Not married	4,678 (33.4)	2,173 (38.9)	2,505 (37.6)	
Educational level				0.619
High school or less	6,699 (49.8)	3,289 (50.4)	3,410 (49.3)	
Some college	2,491 (26.4)	1,157 (25.8)	1,334 (26.9)	
College graduate or higher	1988 (23.8)	955 (23.8)	1,033 (23.7)	
Race and ethnicity				<0.001
Mexican American	1955 (4.0)	984 (4.3)	971 (3.7)	
Non-Hispanic White	6,331 (79.0)	2,653 (74.0)	3,678 (83.3)	
Non-Hispanic Black	1994 (8.7)	1,282 (12.1)	712 (5.8)	
Other	925 (8.3)	497 (9.5)	428 (7.2)	
Family income^a^				0.274
Low	2,742 (17.6)	1,337 (17.6)	1,405 (17.5)	
Medium	4,125 (37.6)	1947 (36.6)	2,178 (38.4)	
High	3,310 (44.9)	1,630 (45.8)	1,680 (44.1)	
Smoking status^b^				0.002
Never	5,217 (46.2)	2,616 (48.1)	2,601 (44.7)	
Former	4,197 (37.1)	2005 (36.9)	2,192 (37.2)	
Current	1771 (16.7)	784 (15.0)	987 (18.1)	
Alcohol drinker^c^				0.636
Yes	7,417 (66.5)	3,563 (66.2)	3,854 (66.7)	
No	3,788 (33.5)	1853 (33.8)	1935 (33.3)	
Hypertension				0.008
Yes	5,730 (47.9)	2,685 (46.3)	3,045 (49.4)	
No	5,475 (52.1)	2,731 (53.7)	2,744 (50.6)	
Hyperlipidemia				0.993
Yes	4,822 (51.2)	2,320 (51.2)	2,502 (51.2)	
No	6,383 (48.8)	3,096 (48.8)	3,287 (48.8)	
Diabetes mellitus				0.469
Yes	2029 (14.1)	985 (14.3)	1,044 (13.8)	
No	9,176 (85.9)	4,431 (85.7)	4,745 (86.2)	
Cataract^d^				<0.001
Yes	2,131 (15.2)	877 (13.0)	1,254 (17.0)	
No	9,074 (84.8)	4,539 (87.0)	4,535 (83.0)	

Data are presented as unweighted number (weighted percentage) unless otherwise indicated.

a Categorized into the following 3 levels based on the family poverty income ratio: low income (≤1.3), medium income (>1.3 to 3.5), and high income (>3.5).

b We categorized smoking status into the following 3 groups: never smoked (smoked <100 cigarettes in life), former smoker (smoked at least 100 cigarettes in life but has quit), and current smoker (smoked at least 100 cigarettes in life and is now still smoking).

c Determined by answering the following question: “In any 1 year, have you had at least 12 drinks of any type of alcoholic beverage?”.

d Cataract operation was determined by asking participants the question, “Have you ever had a cataract operation?” (VIQ070:1999–2002; VIQ071:2003–2008), with answers “yes” or “no.” If the answer was “yes,” the participant was diagnosed with a cataract.

As shown in Table 2, logistic regression analysis found no significant difference between SII and cataract after adjusting for covariates in the whole population. In the women subgroup, the univariate and multivariate analyses demonstrated high SII over 500 × 10^9^/L was associated with a higher risk of cataract in the crude model (OR, 1.40; 95% CI, 1.18–1.66; *p* < 0.001) and model 1 (OR, 1.27; 95% CI, 1.03–1.56; *p* = 0.024). After adjusting for all confounding factors, high SII over 500 × 10^9^/L was still positively associated with cataract among women (OR, 1.27; 95% CI, 1.02–1.59; *p* = 0.036). No difference was found in the men subgroup, and there was no significant interaction between SII and sex (*P* for interaction = 0.198). Because the percentage of missing data was small for any variable in this cross-sectional study, no imputation method was used.

**Table 2:** Univariate and multivariate analyses by the activity-stratified logistic regression model, weighted.

	SII (10^9^/L)		*p*-value	P for interaction
	< 500 (OR, 95% CI)	≥ 500 (OR, 95% CI)		
Overall				
Crude model^a^	1.0 (Reference)	1.37 (1.21–1.55)	<0.001	
Model 1^b^	1.0 (Reference)	1.20 (1.03–1.40)	0.022	
Model 2^c^	1.0 (Reference)	1.16 (0.98–1.38)	0.076	
Sex				0.198
Female				
Crude model	1.0 (Reference)	1.40 (1.18–1.66)	<0.001	
Model 1	1.0 (Reference)	1.27 (1.03–1.56)	0.024	
Model 2	1.0 (Reference)	1.27 (1.02–1.59)	0.036	
Male				
Crude model	1.0 (Reference)	1.30 (1.08–1.57)	0.005	
Model 1	1.0 (Reference)	1.12 (0.90–1.40)	0.299	
Model 2	1.0 (Reference)	1.06 (0.83–1.34)	0.642	

CI, confidence interval; OR, odds ratio; SII, systemic immune-inflammation index. Table shows that logistic regression revealed no significant association between SII and cataracts in the overall population after adjusting for covariates. In the female subgroup, high SII was associated with an increased risk of cataracts in adjusted models. No significant association was found in the male subgroup, and there was no significant interaction between SII and gender.

a Crude model: adjusted for none.

b Adjusted for sociodemographic variables (age, sex, race and ethnicity, family income, educational level, and marital status) and NHANES cycles.

c Adjusted for sociodemographic variables, NHANES cycles, BMI, alcohol drinking status, smoking status, hypertension, hyperlipidemia, and diabetes mellitus.

## 4 Discussion

Cataract, a common cause of vision impairment worldwide, involves clouding of the eye’s lens. Apart from aging, research suggests a connection between cataract development and chronic inflammation (11). Studies have linked markers like C-reactive protein (CRP) and interleukin 6 (IL-6) to cataract risk, indicating their role in lens opacification (24). Inflammation and oxidative stress, typical in chronic inflammation, are positively associated with cataract formation (25–28). Conditions like systemic lupus erythematosus, linked to increased cataract risk, exhibit inflammatory processes that may contribute to cataract formation (29). Research has shown that cataracts are more prevalent in women than in men, with females exhibiting higher rates of lens opacities, especially cortical cataracts (30–32). This disparity is not solely due to higher cataract extraction rates among women in Western countries, and there is currently no definitive evidence linking lifestyle-related factors to this gender difference.

SII was calculated by neutrophils, lymphocytes, and platelets. It reflects the inflammatory reactions and may be a useful diagnostic biomarker for systemic inflammatory activity. Previous studies have found that the SII is associated with PCME in patients without risk factors following uneventful phacoemulsification cataract surgery, suggesting that SII could serve as a predictive biomarker for PCME (9). Additionally, research shows that a pro-inflammatory diet, as measured by the DII, is linked to an increased risk of cataract and AMD, with inflammatory markers such as the NLR mediating these associations (11). However, there is currently a gap in large-scale studies directly investigating the relationship between SII and cataracts. Our results indicated that a SII higher than 500 × 10^9^/L was positively correlated with an increased risk of cataracts in women. Therefore, SII with a non-intrusive methodology and low cost can be identified as a biomarker for cataract among women.

Our study examined the link between SII and cataracts specifically in women and found that elevated SII levels were associated with cataracts. This suggests that anti-inflammatory therapies or lifestyle changes could be beneficial for those with chronic inflammation. The evidence highlights the need for further research to better understand this relationship and improve cataract prevention and treatment. Additionally, our findings indicate that higher SII levels are associated with current smoking, suggesting that smoking may contribute to elevated systemic inflammation. This underscores the importance of smoking cessation programs, as reducing smoking could potentially lower inflammation and mitigate associated health risks.

Our research employed a robust methodology with a large and diverse sample size drawn from NHANES data to investigate the relationship between SII and cataracts. By conducting subgroup analyses and carefully adjusting for confounding variables, we minimized bias and increased the statistical accuracy of our findings. This study contributes valuable insights to the existing knowledge base and sheds light on the correlation between elevated SII levels and cataract risk among adult outpatients in the United States.

Based on our findings, SII could be integrated into a broader risk assessment for cataracts, particularly for higher-risk populations. Routine screening might include measuring SII levels along with other established risk factors. For individuals with elevated SII, interventions could involve lifestyle changes such as improved diet, increased physical activity, and smoking cessation, as these can help manage inflammation and oxidative stress, which are modifiable risk factors for cataracts. Enhanced surveillance through more frequent eye examinations may also be recommended for those with high SII to facilitate early detection and management. Future research should investigate whether anti-inflammatory or antioxidant therapies could benefit individuals with elevated SII.

As a cross-sectional study, our ability to establish causal relationships is limited. To address this, future research should explore longitudinal or experimental designs. Additionally, we cannot fully eliminate the potential impact of residual confounding from unmeasured variables, including other inflammatory markers, lifestyle factors such as diet and physical activity, and exposure to environmental toxins. Although NHANES data provide national representativeness, the generalizability of our findings to other countries remains uncertain. Further research is needed to better understand the complexities of the link between chronic inflammation and cataracts, which could lead to innovative preventive measures and treatment options in cataract management.

## 5 Conclusion

In this cross-sectional study involving 11,205 participants from a large nationally representative survey, it was observed that a high SII over 500 × 10^9^/L was positively associated with cataract development among women, even after adjusting for confounding factors. This study contributes valuable insights into the relationship between high SII levels and cataract risk in outpatient adults in the United States. Moving forward, larger prospective cohorts are needed to validate these results, emphasizing the importance of addressing inflammation to mitigate cataract progression and improve patient outcomes.

## Data Availability

Publicly available datasets were analyzed in this study. This data can be found at: https://www.cdc.gov/nchs/nhanes/index.htm.
